# Study of arthroscopic superior capsule reconstruction in the treatment of irreparable rotator cuff tears

**DOI:** 10.3389/fsurg.2022.895571

**Published:** 2023-01-06

**Authors:** Kai Sun, Yijin Li

**Affiliations:** Orthopedics of Tianjin First Center Hospital, Tianjin First Central Hospital, Tianjin, China

**Keywords:** level IV, therapeutic case series arthroscopic, superior capsule reconstruction, irreparable rotator cuff tears, technique, shoulder joint

## Abstract

**Background:**

The objective of this study was to investigate the clinical outcome and radiographic findings after arthroscopic superior capsule reconstruction (ASCR) with a new augmented autograft technique for irreparable rotator cuff tears.

**Methods:**

Between 2018 and 2020, 11 patients whose shoulders had irreparable rotator cuff tears underwent ASCR using a fascia lata weaving mesh. Physical examination, radiography, and magnetic resonance imaging (MRI) were performed before surgery and the average follow-up was 20 months (18–24 months) after surgery. Clinical outcome scores were recorded.

**Results:**

Average clinical outcome scores improved significantly at the final follow-up, with 94.7 points scored on the American Shoulder and Elbow Surgeons scale (range, 85–100 points) and 34.5 points on the University of California, Los Angeles scale (33–35 points) (*P* < 0.05). Mean active elevation increased significantly from 30.1° to 150° (*P* < 0.05) and external rotation increased from 30° to 59.2° (*P* < 0.05). The acromiohumeral distance (AHD) increased from 3.9 ± 0.6 mm preoperatively to 10.1 ± 0.7 mm postoperatively (*P* < 0.05). No patient had graft tear or tendon retear during follow-up.

**Conclusions:**

ASCR with a new augmented autograft can restore the function of the shoulder joint with irreparable rotator cuff tears. Our results suggest that this reconstruction technique can help obtain good clinical and radiographic outcomes, which can provide a reliable method for the treatment of irreparable rotator cuff tears.

**Level of Evidence:**

Level IV, therapeutic case series.

## Background

Chronic large or massive rotator cuff tears are often associated with tendon retraction, fat infiltration, and muscle atrophy, which cannot be repaired by conventional surgery or will be prone to retear after repair (1). It is difficult to repair these massive irreparable rotator cuff tears; moreover, the rate of retear repair is high. Its treatment is controversial, and there is no unified standard treatment (2). The main clinical treatment methods include arthroscopic debridement, long head tendon transection of biceps brachii, partial rotator cuff repair, tendon transfer, patch bridging technology, upper joint capsule reconstruction technology, and reverse shoulder replacement technology (3). The reconstruction of the upper articular capsule can simulate the structure of the shoulder capsule, correct mechanical imbalance, stabilize the glenohumeral joint, maintain the sealing of the articular cavity, and secrete synovial fluid to nourish the cartilage, and all these can help avoid further injury of the shoulder joint. Previously, Mihata et al. made use of the fascia lata as a patch for reconstructing the upper joint capsule and used arthroscopy combined with small incision technology (4). In contrast, we adopted a new augmented autograft in the management of irreparable posterosuperior rotator cuff tears. Here, we report the results of our technique.

## Methods

### Patient demographics

We retrospectively reviewed our database, which was prepared prospectively. Between 2018 and 2020, 11 patients with irreparable rotator cuff tears (5 large, 6 massive) (a torn tendon cannot reach the original footprint during shoulder arthroscopy) were managed with arthroscopic superior capsule reconstruction (ASCR). The exclusion criteria included severe bone deformity such as Hamada classification type V, Goutallier 3–4, cervical nerve palsy, axillary nerve palsy, deltoid muscle dysfunction, and infection. Consequently, 11 patients whose shoulders had irreparable rotator cuff tears were enrolled in the study. Of these, there were 4 females and 7 males with an average age of 60.7 years (Table 1). The patients signed an informed consent form approved by the Institutional Review Board at our hospital (Tianjin First Center Hospital). We measured active shoulder elevation, external rotation, and internal rotation and assessed all patients preoperatively and at final follow-up (18–24 months) after surgery by using the scoring systems of the shoulder index of the American Shoulder and Elbow Surgeons (ASES, a 100-point scoring system) (5), University of California, Los Angeles (UCLA, a 35-point scaling system) (6), and acromiohumeral distance (AHD).

**Table 1:** Summary of patients.

Shoulder	Sex	Age (year)	Occupation	Duration of symptoms (month)	Tear size	Hamada classification (stage)	Torn tendon	Operation
1	M	55	Manual worker	10	Large	2	SubS, ISP	Primary
2	F	59	Housewife	12	Massive	2	SubS, SSP	Primary
3	M	61	Manual worker	15	Large	2	SSP, ISP	Primary
4	M	58	Manual worker	18	Massive	1	SubS, SSP	Primary
5	M	59	Farmer	13	Massive	2	SubS, SSP	Primary
6	F	64	Housewife	8	Large	2	SubS, SSP	Primary
7	F	61	Housewife	10	Massive	1	SubS, SSP	Primary
8	M	72	Manual worker	14	Massive	2	SubS, SSP	Primary
9	M	60	Farmer	18	Massive	2	SubS, SSP	Primary
10	M	58	Farmer	18	Large	2	SSP, ISP	Primary
11	F	59	Housewife	16	Large	2	SSP, ISP	Primary

M, male; F, female; ISP, infraspinatus; SSP, supraspinatus; SubS, subscapularis; primary, primary surgery.

### Radiography and magnetic resonance imaging

**Figure 1:**
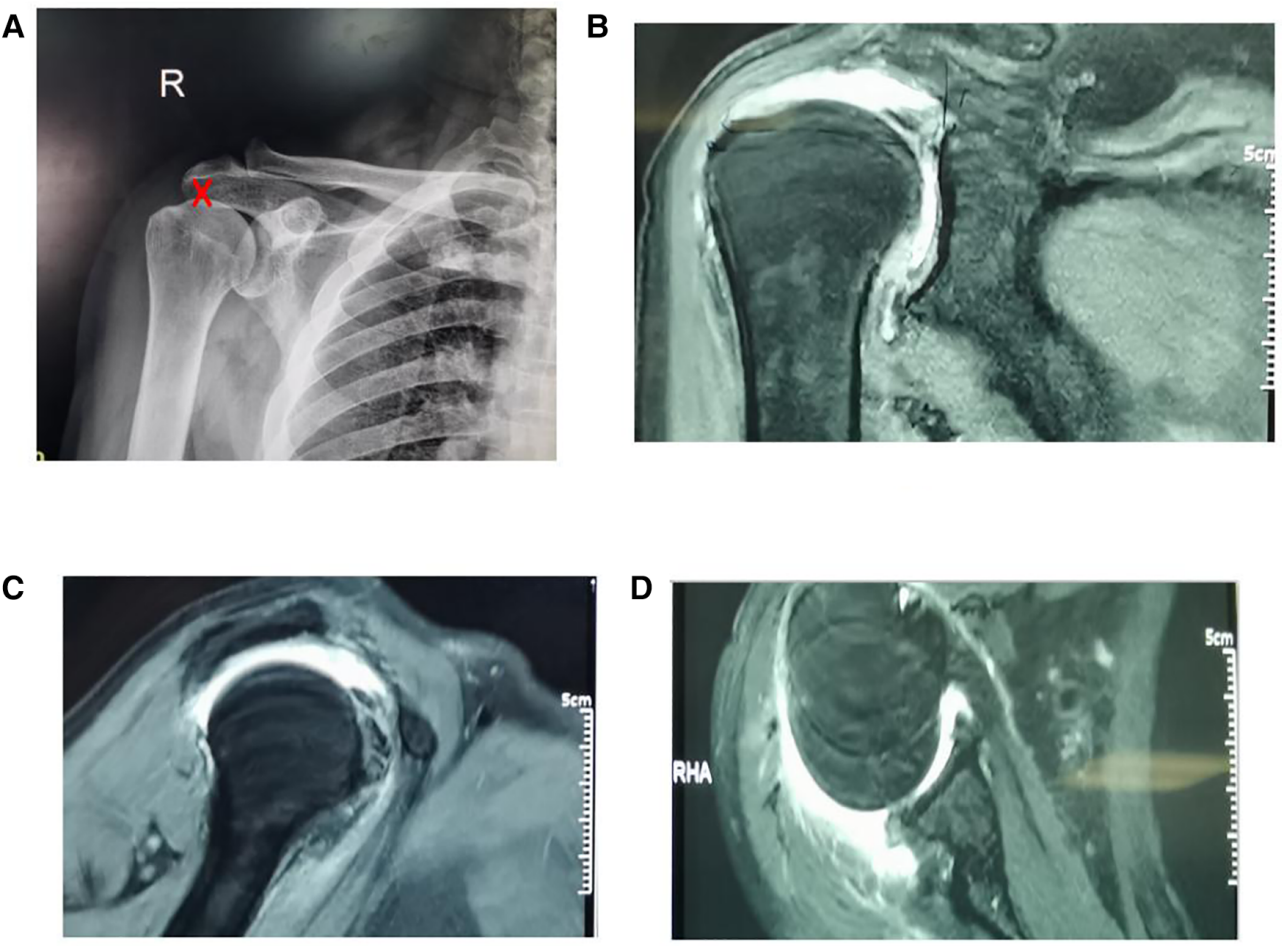
Preoperative radiograph of irreparable rotator cuff tears. (**A**) Red mark on the X-ray shows that the acromiohumeral distance (AHD) is 3.5 mm; (**B–D**) show huge irreparable rotator cuff tears.

We obtained preoperative (Figures 1A–D) and follow-up radiographs in all patients. According to the method described by Ellman et al., we measured the AHD (7). Fatty degeneration of the rotator cuff was evaluated using the method described by Goutallier et al. (8). Radiography was performed before and after surgery at 3, 6, 12, and final follow-up months by the surgeon.

### Surgical technique

**Figure 2:**
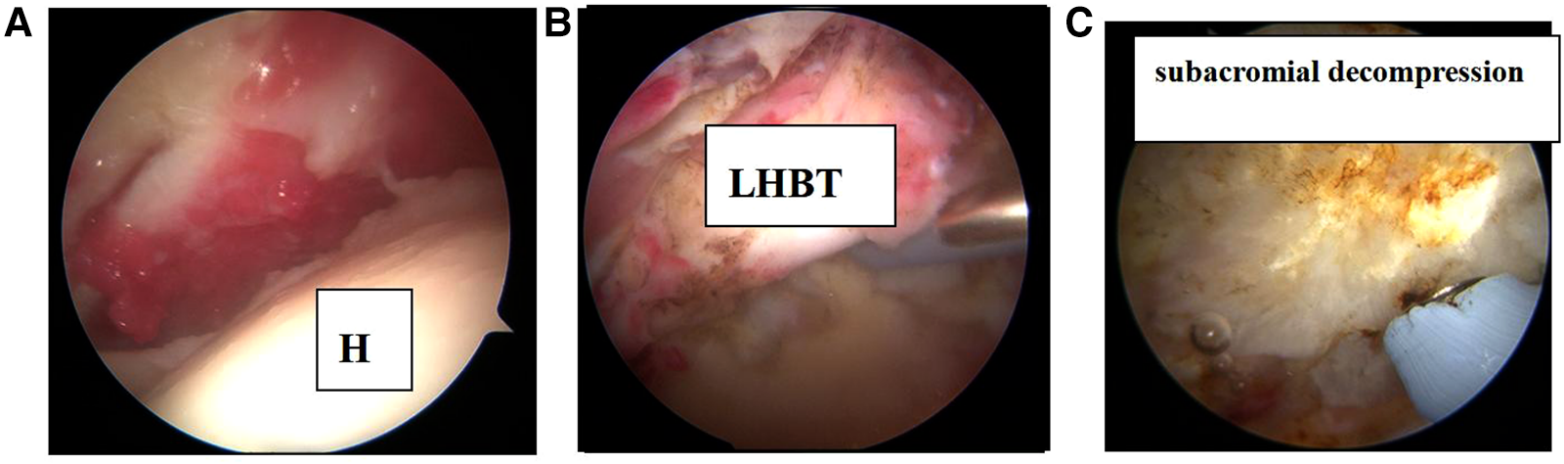
Arthroscopic images of the rotator cuff tear in the right shoulder in patient 2. (**A**) Irreparable rotator cuff tears; (**B**) rupture of the long head of the biceps brachii tendon; (**C**) arthroscopic subacromial decompression to create a flat acromial undersurface. H, humeral head; LHBT, long head of the biceps brachii tendon.

The patients were placed in the beach chair position using general anesthesia. We measured the shoulder range of motion and laxity. The posterior, anterior, anterolateral, and lateral portals were established. We cleared the joint cavity, explored the type of rotator cuff injury, removed the long head of the biceps brachii tendon (Figures 2A,B), and then performed subacromial decompression (Figure 2C). If the supraspinatus muscle cannot be pulled to the anatomical footprint after release, this means that the supraspinatus muscle cannot be repaired. The long head of the biceps brachii tendon was ruptured or fixed. The anterior and posterior diameters and internal and external diameters of the supraspinatus muscle defect were measured at 45° shoulder abduction.

**Figure 3:**
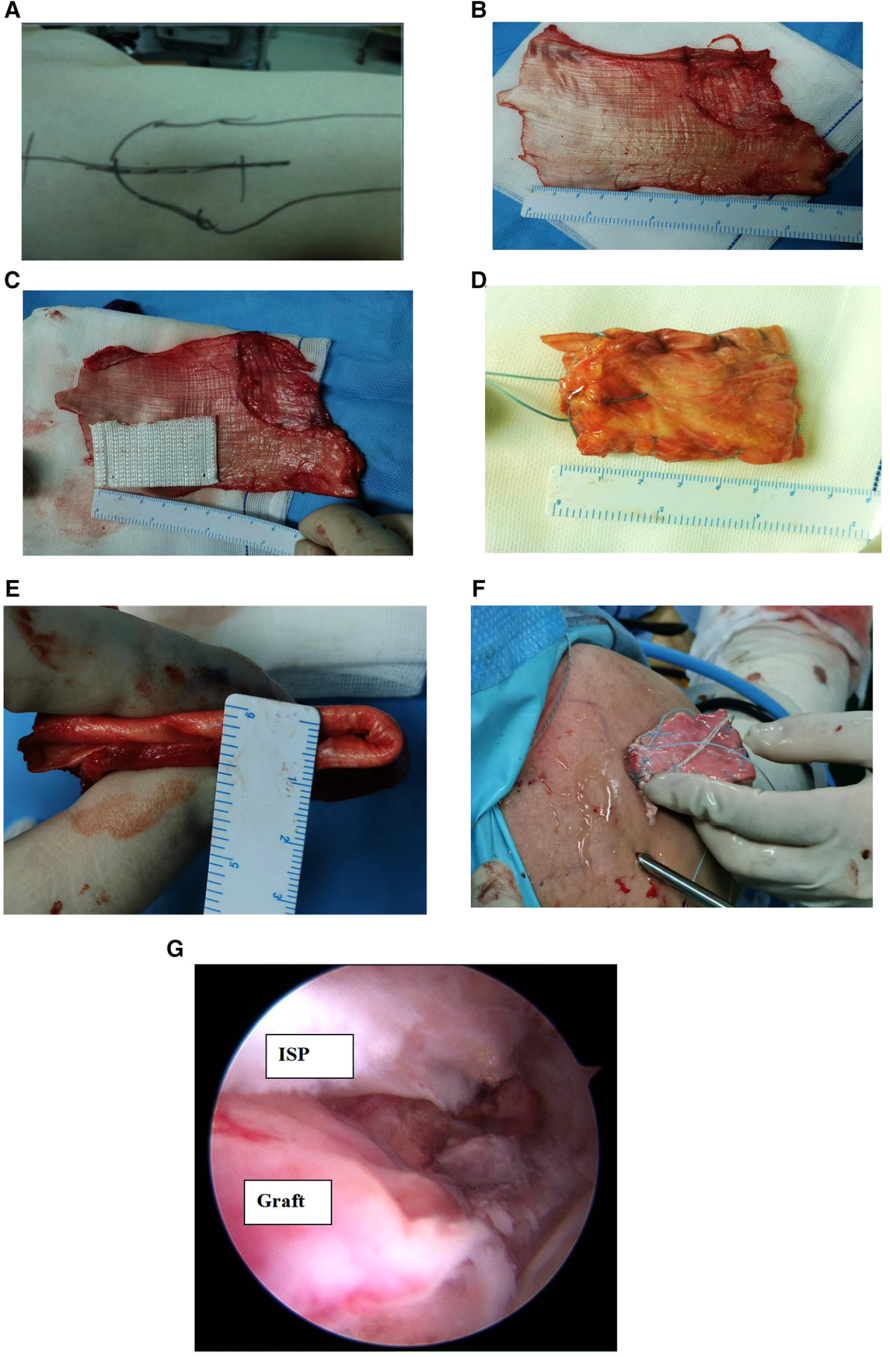
Intraoperative radiograph of arthroscopic superior capsule reconstruction. (**A**) Vertical skin incision; (**B**) harvested section of the fascia lata, two times the size of the superior capsular defect; (**C**) folding the fascia lata in half, combined with a pet material; (**D, E**) weaving the four sides with a non-absorbable suture to make a fascia lata patch, on which a graft 5.5–6.0 cm in length and 6–8 mm in thickness was fashioned by folding the fascia lata twice thick; (**F**) insertion of the fascia lata into the subacromial space through the anterolateral portal; (G) arthroscopic superior capsule reconstruction. ISP, infraspinatus tendon.

We made a 12 cm lateral longitudinal incision (Figure 3A) at the proximal of the ipsilateral thigh and harvested a section of the fascia lata (Figure 3B). The length was approximately twice the internal and external diameter of the rotator cuff defect, and the width was approximately the anterior and posterior diameter of the defect. We folded the fascia lata in half, combined it with a pet material (Figure 3C), and weaved the four sides with a non-absorbable suture to make a fascia lata patch, on which we fashioned a graft 5.5–6.0 cm in length and 6–8 mm in thickness by folding the fascia lata twice thick (Figures 3D,E). We inserted the fascia lata into the subacromial space through the anterolateral portal (Figure 3F) and attached the medial side to the superior glenoid at 10–11 o'clock and 11–12 o'clock positions of the right shoulder or at 1–2 o'clock and 12–1 o'clock positions of the left shoulder by using a glenoid lip anchor (diameter, 5 mm, Arthrex). The medial side was fixed to the supraspinatus muscle, the lateral side was fixed to the greater tuberosity of freshened rotator cuff footprints by double row fixation, the anterior side was sutured to the rotator cuff gap tissue, and the posterior side was sutured to the infraspinatus muscle at 45° shoulder abduction (Figure 3G).

### Postoperative protocol

A pillow was abducted 30° and fixed for 4 weeks. Passive- and active-assisted exercise and strength training were allowed 8 weeks after surgery, and there was no restriction on activity for 1 year after operation. Physical therapists assisted all patients.

### Statistical analysis

Physical examination and radiography imaging were performed after surgery and yearly thereafter. The average follow-up was 20 months (18–24 months) after surgery. We compared the shoulder range of motion, ASES, and UCLA at final follow-up. All data were reported as the mean ± standard deviation. Values were compared using multiple comparisons, where *P*-values of 0.05 or less were considered statistically significant.

## Results

### Clinical results

We obtained preoperative and follow-up radiographs in all patients. The average follow-up was 20 months (18–24 months) after surgery. The average preoperative scores were 11.7 points on the ASES scale (range, 5–25 points) and 13.7 points on the UCLA scale (9–18 points). Average clinical outcome scores improved significantly at the final follow-up after surgery: 94.7 points on the ASES scale (range, 85–100 points) and 34.5 points on the UCLA scale (33–35 points) (*P* < 0.05) (Table 2). All workers and farmers among the patients had returned to work at the final follow-up. The active range of shoulder motion improved significantly, with 119.9° for elevation (*P* < 0.05), 29.2° for external rotation, and by four vertebral bodies for internal rotation (Table 3). There were no cases of infection, neural injury, or suture anchor problems.

**Table 2:** Summary of patients'shoulder functional scores.

Shoulder	ASES (scores)		UCLA (scores)		AHD (mm)	
	Preoperative	Postoperative	Preoperative	Postoperative	Preoperative	Postoperative
1	25	100	18	35	4.1	10.1
2	15	100	15	35	4.3	9.8
3	13	100	12	34	4.2	10.5
4	20	95	16	35	5.1	11.2
5	5	95	12	35	3.5	9.5
6	10	90	17	35	4.5	11.3
7	8	95	15	34	4.0	10.8
8	10	90	11	33	3.5	9.8
9	5	95	12	35	3.5	10.1
10	8	90	14	35	3.0	9.1
11	10	85	9	33	3.0	9.4
Average	11.7	94.7	13.7	34.5	3.9	10.1
SD	5.9	4.7	2.27	0.8	0.6	0.7

ASES, American Shoulder and Elbow Surgeons (a 100-point scoring system); UCLA, University of California, Los Angeles (a 35-point scaling system); AHD, acromiohumeral distance (AHD).

**Table 3:** Summary of patients' shoulder range of motion.

Shoulder	Active elevation (°)		Active External Rotation (°)		Active Internal Rotation (°)	
	Preoperative	Postoperative	Preoperative	Postoperative	Preoperative	Postoperative
1	20	160	20	60	S	T12
2	30	160	30	60	S	T10
3	20	130	20	50	L5	T12
3	30	140	30	60	S	T12
4	30	150	40	60	L4	T10
5	40	160	30	60	S	T12
6	20	140	20	50	S	L1
7	40	160	30	50	L5	L1
8	50	170	30	60	S	T12
9	30	150	30	60	L5	T12
10	40	150	40	90	S	L1
11	30	150	40	50	L5	T12
Average	30.1	150	30	59.2	L5	T12
SD	9.0	10.8	7.1	10.4	–	–

Postoperative designates the final follow-up (mean, 20 months; range, 18–24 months after surgery). SD, standard deviation.

### Radiographic evaluation

**Figure 4:**
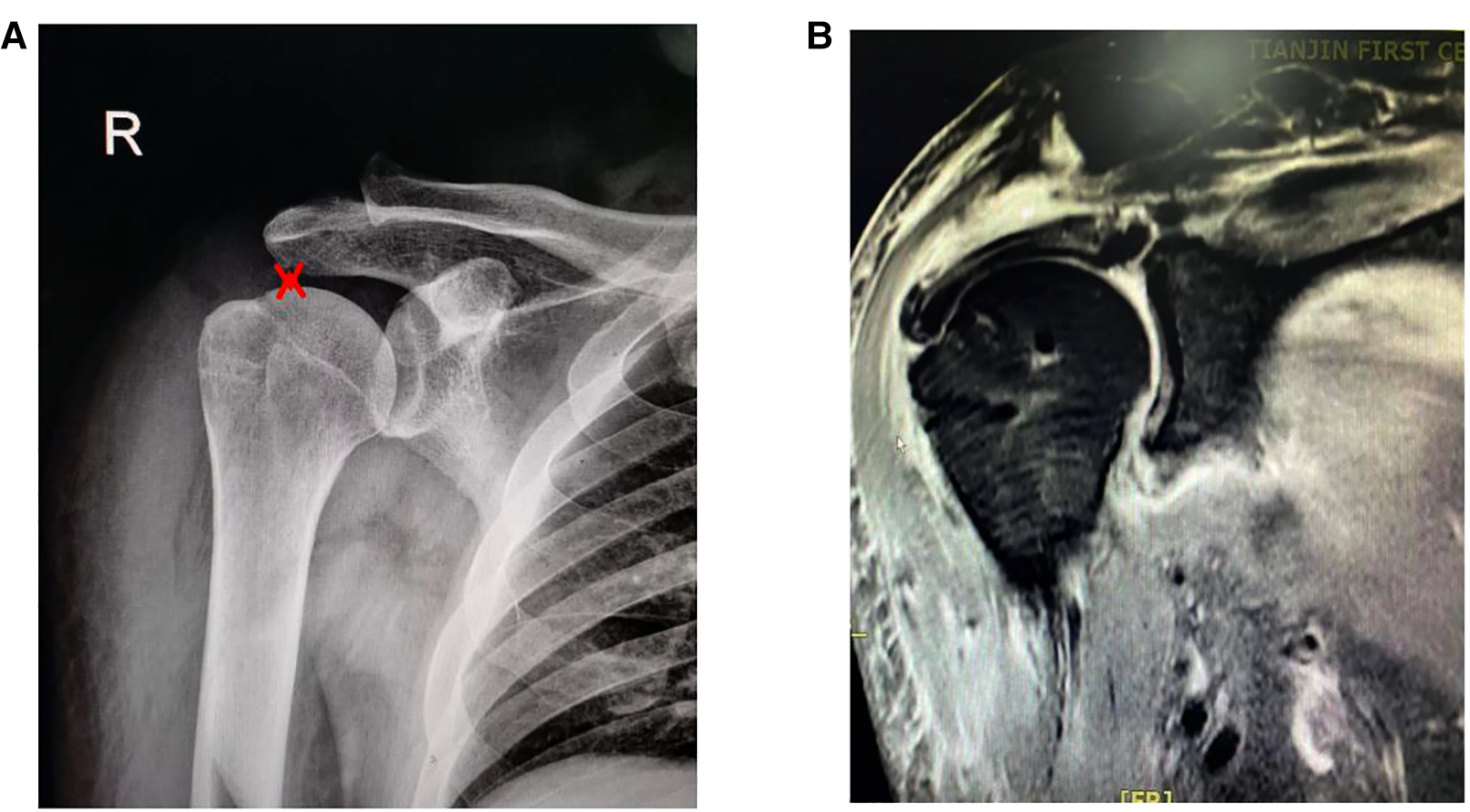
Postoperative radiograph of arthroscopic superior capsule reconstruction (1 month). (**A**) Red mark on the X-ray shows that the AHD is 9.5 mm; (**B**) the fascia lata medial side attached to the superior glenoid at 10–11 o'clock and 11–12 o'clock positions in the right shoulder, using two fully-threaded titanium suture anchors (diameter, 5 mm; suture anchor, Arthrex).

**Figure 5:**
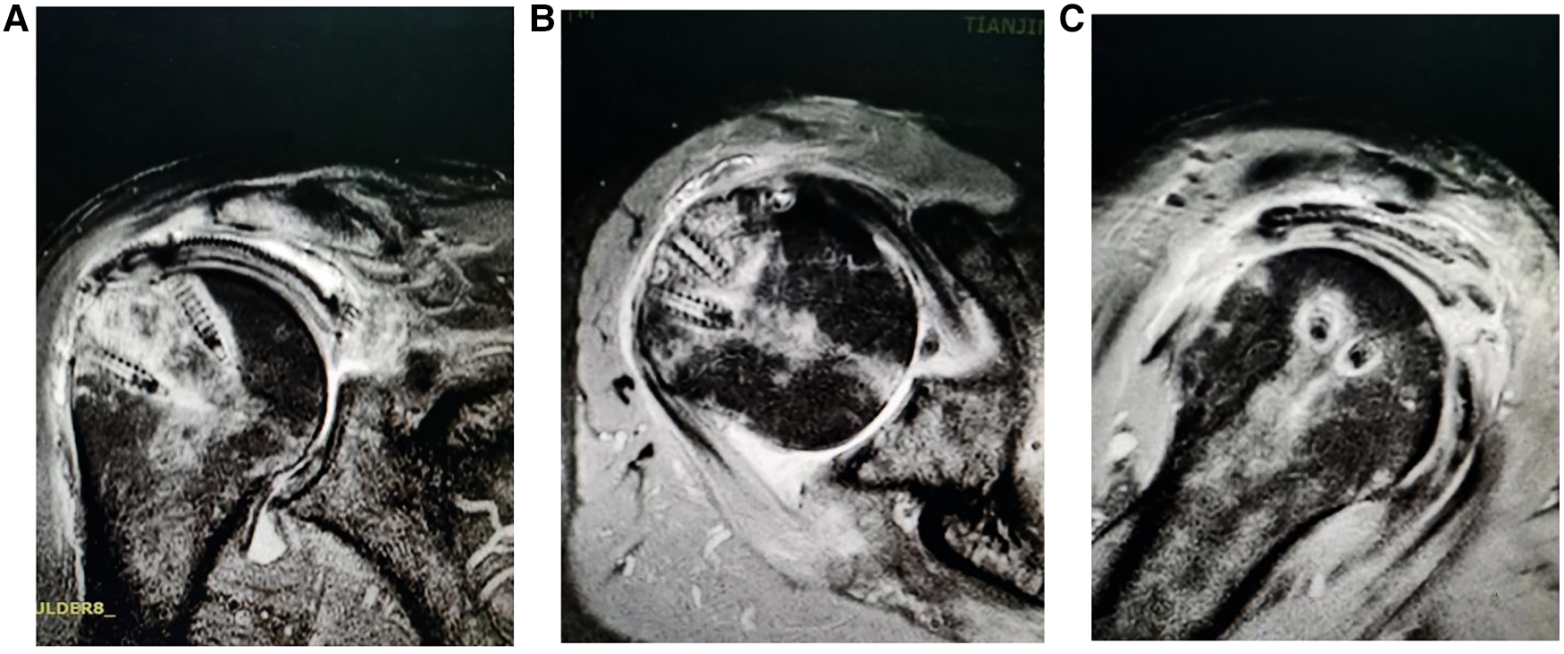
Postoperative radiograph of arthroscopic superior capsule reconstruction (20 months). (**A, B**) The fascia lata lateral side is fixed to the rotator cuff footprints on the greater tuberosity using double row fixation and (**C**) a completely healed fascia lata.

The preoperative AHD was 3.9 ± 0.6 mm and 10.1 ± 0.7 mm at the final follow-up and improved significantly (Figure 4A) (*P* < 0.05). At the final follow-up magnetic resonance imaging (MRI), all fasciae latae had completely healed (Figures 4B, 5A–C). In this study, 11 patients with subscapular muscle injury were healed after 20 months follow-up. In accordance with the exclusion criteria, the fatty degeneration of the infraspinatus muscle and infraspinatus tendon was not measured and muscle atrophy was not shown in this study.

## Discussion

Irreparable rotator cuff tear is often accompanied by fat infiltration, tendon retraction, muscle atrophy, and so on. The reparative operation is complex and the failure rate is very high (9). Although a variety of surgical methods have been reported, including tendon transposition, patch bridging, superior capsule reconstruction, and reverse shoulder replacement, there is no unified standard treatment (10–15). Many patients with irreparable rotator cuff tears have difficulty in fully pulling back the residual rotator cuff tissue to the footprints. Some scholars have reported the use of partial repair technology. Yoo et al. (16) observed the integrity of the rotator cuff with MRI after partial repair and found that the retear rate was 45.5%. Berth et al. (4) conducted a clinical randomized controlled trial and found that there was no difference between the curative effect of partial repair and simple joint cleaning, and the partial repair retear rate was as high as 52% (17, 18). Tendon transposition surgery, such as Chinese way technology, is an excellent shoulder static stabilizer and subacromial space spacer, saving materials and internal fixation consumables. However, tendon transfer is relatively controversial, and latissimus dorsi tendon transfer has been questioned (19). Therefore, it is difficult to popularize these in the clinic because of their high postoperative retear rate.

Superior capsule reconstruction refers to the technique of reconstructing and restoring the integrity of the rotator cuff by using patch material to anchor the glenoid medially and fix the lateral part in the footprints of the greater tuberosity (19). Its advantage is the restoration of the anatomical integrity of the rotator cuff, and its clinical effect is related to a variety of objective factors, including the selection of patch materials, surgical technology, inclusion criteria (degree of rotator cuff fat infiltration), etc. Early studies have shown that the retear rate of an artificial patch is 62% (20).

A study (21) reported that the AHD did not change significantly after ASCR in irreparable rotator cuff tear. Clinical results showed that superior stability, destroyed by large rotator cuff tears, cannot be restored. However, our clinical outcome showed that the AHD significantly increased, and preoperative AHD was 3.9 ± 0.6 mm and 10.1 ± 0.7 mm at the final follow-up after ASCR. Furthermore, there were no graft tears or retears at the final follow-up. Therefore, we opine that superior capsule reconstruction is implementable and reliable.

In addition, the degree of fat infiltration in the rotator cuff has an important impact on the clinical effects. Mori et al. (21) reported that autologous fascia lata patch bridging and partial repair technology were used to treat patients with huge rotator cuff tear, with low fat infiltration observed. It was found that the clinical effect of the patch bridging group was significantly better than that of the partial repair group, and the retear rate of the infraspinatus muscle decreased from 41.7% to 8.3% in the partial repair group. However, in subsequent clinical studies, the authors found that in patients with high fat infiltration (Goutallier grade 3–4), the retear rate was still as high as 89.4% (22), and its effect was similar to that of partial repair. Thus, the lower the degree of fat infiltration, the better the treatment effect. Therefore, standard ASCR may not be a good treatment method in irreparable rotator cuff tear with severe fatty degeneration. Hence, severe bone deformity and Goutallier grades 3–4 were excluded.

Most surgical treatments such as Chinese way and other tendon transposition methods can decrease shoulder pain (23, 24), but it is difficult to restore muscle strength, range of motion and active elevation, and external rotation. In our patients, the average preoperative scores were 11.7 and 13.7 points on the ASES and UCLA scales, respectively. Average clinical outcome scores improved significantly at the final follow-up after surgery; 94.7 and 34.5 points on the ASES and UCLA scales, respectively. All workers and farmers among these patients had returned to work at the final follow-up. The shoulder active range of motion improved significantly.

We suggest that ASCR technology can help restore the original physiological and anatomical structure, and it is theoretically an ideal operation for the treatment of irreparable rotator cuff tears. Mihata et al. (23) conducted cadaveric biomechanical research and found that when the supraspinatus muscle and superior capsule were damaged at the same time, the humeral head translated upward, increasing from 3.7 to 5.4 mm, and the subacromial pressure increased from 0.05 to 0.07 MPa. After reconstruction, the humeral head and subacromial pressure decreased significantly, to only 3.0 mm and 0.01 MPa. Therefore, the authors suggest that ASCR is an important anatomical procedure to maintain stability of the humeral head (25). Subsequently, the clinical study conducted by Mihata et al. also presented this conclusion.

### Limitations

This study has some shortcomings. First, this study is only a retrospective series analysis. The number of cases is small, and we did not compare ASCR with other operation treatments. There is a certain bias in the conclusion. Second, this study presents only a short-term follow-up. Although the patients’ patch had healed, it is still unclear whether it can stabilize the humeral head for a long time, and further follow-up is needed to study the long-term effect. In a future study, we intend to use a control group to draw comparisons between ASCR and Chinese way, or other forms of arthroscopic rotator cuff repair, so that long-term effects can be established in more patients after follow-up.

## Conclusions

ASCR with a new augmented autograft can restore superior glenohumeral stability for the treatment of irreparable rotator cuff tears. Our results suggest that this technique is reliable and useful.

## Data Availability

The raw data supporting the conclusions of this article will be made available by the authors, without undue reservation.
